# A comparison of two mathematical models of the impact of mass drug administration on the transmission and control of schistosomiasis

**DOI:** 10.1016/j.epidem.2017.02.003

**Published:** 2017-03

**Authors:** J.E. Truscott, D. Gurarie, R. Alsallaq, J. Toor, N. Yoon, S.H. Farrell, H.C. Turner, A.E. Phillips, H.O. Aurelio, J. Ferro, C.H. King, R.M. Anderson

**Affiliations:** a London Centre for Neglected Tropical Disease Research, Department of Infectious Disease Epidemiology, Imperial College, Norfolk Place, St. Mary’s Campus, London, UK; b Schistosomiasis Control Initiative, Department of Infectious Disease Epidemiology, Imperial College, Norfolk Place, St. Mary’s Campus, London, UK; c Center for Global Health and Diseases, Case Western Reserve University, 10900 Euclid Avenue LC: 4983, Cleveland, OH 44106, United States; d Department of Mathematics, Case Western Reserve University, 10900 Euclid Avenue LC: 4983, Cleveland, OH 44106, United States; e Universidade Catholica de Moçambique, Beira, Mozambique

**Keywords:** Schistosomiasis, Mass drug administration, Neglected tropical diseases, Mathematical modelling

## Abstract

The predictions of two mathematical models describing the transmission dynamics of schistosome infection and the impact of mass drug administration are compared. The models differ in their description of the dynamics of the parasites within the host population and in their representation of the stages of the parasite lifecycle outside of the host. Key parameters are estimated from data collected in northern Mozambique from 2011 to 2015. This type of data set is valuable for model validation as treatment prior to the study was minimal. Predictions from both models are compared with each other and with epidemiological observations. Both models have difficulty matching both the intensity and prevalence of disease in the datasets and are only partially successful at predicting the impact of treatment. The models also differ from each other in their predictions, both quantitatively and qualitatively, of the long-term impact of 10 years’ school-based mass drug administration. We trace the dynamical differences back to basic assumptions about worm aggregation, force of infection and the dynamics of the parasite in the snail population in the two models and suggest data which could discriminate between them. We also discuss limitations with the datasets used and ways in which data collection could be improved.

## Highlights

• This paper compares two mathematical models describing the transmission dynamics of schistosome infection and the impact of mass drug administration.
• The models differ structurally in a number of ways, including the dynamics of the intermediate snail host and the treatment of adult worms within the human host.
• The models are validated against data taken from a mass-drug administration trial in Mozambique.
• The differences between the model predictions and the data are discussed in the context of the structural differences between the models.

## 1 Introduction

Mathematical and computational tools are essential for synthesizing information to understand epidemiological patterns, and for developing and weighing the evidence base for decision-making in public health policy. The field of mathematical model development for the study of infectious disease epidemiology and control has been recently reviewed by Heesterbeek and colleagues (Heesterbeek et al., 2015). They document many examples where models have been influential in the formulation of public health policy, especially for directly transmitted viruses such as influenza A.

In recent years, efforts have been made to compare the behaviour of independently developed models, parameterized with standardized data, in order to understand the sometimes substantial differences between their predictions. A prime example is the HIV modelling consortium, which has had considerable success in understanding the key features of HIV transmission and identifying important biases in existing models (Eaton et al., 2015). More formal, Bayesian methods are also being employed to create weighted model ensembles which can generate more robust predictions of epidemic progress (Lindström et al., 2015, Webb et al., 2017). The Bill and Melinda Gates Foundation (BMGF) recently funded a consortium of research groups to develop mathematical models of the transmission dynamics and impact of control measures of certain neglected tropical diseases (NTDs) (http://www.ntdmodelling.org). Two groups were funded to address each of the chosen infectious diseases to allow comparisons of the predictions of different mathematical models relating to how various control measures impact the prevalence and intensity of infection in defined settings (Anderson et al., 2015, Gurarie et al., 2015, Hollingsworth et al., 2015).

Studies of the transmission dynamics of the *Schistosoma* infections have a long history, dating back to the pioneering work of George Macdonald. His models were the first to use differential equations with mating probabilities to represent the dioecious nature of these parasites and the fact that they cannot leave the host in the adult worm state to find a mate (Macdonald, 1965). This is critical, as both female and male worms must be present in the same host to create viable eggs to continue the parasite life cycle. Prior to Macdonald’s studies, Nelson Hairston had employed life table analyses from population ecology in an attempt to quantify reproduction and mortality throughout the two-host life cycle of these digenean parasites (Hairston, 1962, Hairston, 1965). More recent work in this field has been reviewed by Anderson and colleagues (Anderson et al., 2016).

The present paper considers two mathematical models to describe the transmission dynamics and control of the schistosome parasites. One model is deterministic in structure, dividing the host population into classes harbouring different burdens of parasites (as originally described by Kostitzin, 1934) and includes greater detail on the parasite life cycle, including the dynamics of the intermediate snail host (Gurarie et al., 2015). The second model considers the dynamics of the adult worm in the human host, with a hybrid structure representing the probability distribution of parasites per host and the associated mating function (for a negative binomial distribution with fixed value *k* and monogamous mating) (Anderson et al., 2015). These two models have some similarities, however, there are significant differences in the structure and methods employed in parameter estimation. These differences are described in brief in the Methods section.

Model validation is based on a data set from a study of targeted mass-drug administration (MDA; targeted by age group) strategies performed in northern Mozambique from 2011 to 2015. This data set is particularly valuable for model validation as it was carried out in a population that had not previously been heavily treated. Formal quantitative comparisons are made on the impact of a defined MDA programme on infection prevalence and intensity over time.

## 2 Methods

### 2.1 Imperial college London (ICL) model

The model developed by the ICL team has an age structured deterministic partial differential framework with probability elements for the description of parasite natural history and transmission (Anderson et al., 2015). Partial differential equations representing changes over time and age are employed to describe the evolution of the mean worm burden (MWB) in an age-structured population and the dynamics of a single shared environmental reservoir of infection from the cercarial stage released by the snail intermediate host (See Supplementary Information 1). It assumes that the parasite is dioecious and monogamous, has density-dependent egg production and a degree of parasite aggregation across hosts defined by the negative binomial (NB) probability distribution with a fixed *k* value. A stochastic individual based analogue of this model has recently been described where the parasite distribution within the human host population is dynamic over time and age group, but the mean predictions of replicated runs of the stochastic model well match the deterministic predictions described in this paper (Farrell et al., 2017). In the model, it is assumed that the age-intensity profiles are generated by age-dependent exposure to the aquatic cercarial stages in the environment and not through acquired immunity. Though the model has a continuous age structure, the outputs can be grouped into programmatically meaningful categories (such as school-aged children; SAC 5–14 years of age and adults ≥15 years of age) which form the basis of the individuals to be targeted in a treatment programme.

### 2.2 Case western reserve university (CWRU) model

The CWRU model employs a stratified worm burden (SWB) approach based on the model originally developed by Kostitzin (1934) for age-structured host communities (Gurarie et al., 2010). In this approach, each community and population group is subdivided into worm burden strata with transitions among strata determined by the rate of worm accumulation, i.e. human force of infection (FOI, λ), and worm mortality (natural or drug-induced). The system accommodates essential features of in-host biology: worm mating, density-dependent reduction in fecundity, and irregular (over-dispersed) egg release from tissue into human urine or stool. The worm distribution within hosts is not constrained by any prescribed function, and the resulting patterns are typically Poisson-like. To account for overdispersed test results it is assumed that the egg output by fertilized female worms and individual hosts in the CWRU model follows a negative binomial distribution with small aggregation parameter *k* (see Gurarie et al., 2016, Hubbard et al., 2002).

Each demographic group has a specific set of parameters (FOI λ, maximal fecundity, density-dependent loss in fecundity and aggregation *k*). The model predicts egg release by individual hosts, their worm burden strata and aggregate host communities as functions of age-dependent FOIs and in-host (biological) parameters. The egg-release function serves a double role. It allows simulation of the outcome of a typical diagnostic test for sampled groups and communities, as is used in model calibration. It also gives estimates of the force of snail infection, and thereby gives transmission coefficients (“human-to-snail” and “snail-to-human”) in coupled human-snail systems. The snail model utilizes a logistic model of population growth and SEI infection with susceptible [S], prepatent [exposed; E] and patent [infected; I] compartments, with known or calibrated local environmental carrying capacity, snail growth, death and proliferation, and estimated patency for transmission. An important novel feature of snail system is nonlinear(saturated) FOI as functions of human infectivity. The complete human-snail model is a deterministic system of differential equations, with the host population stratified according to age and burden, with a corresponding 3-compartment snail component.

For the modelled Mozambique communities, the demographic categorization consisted of 3 age groups: Pre-SAC 0–5 years of age, SAC 5–14 years of age, and adults >15 years of age, where each group was represented by its own SWB with age-specific parameters. A complete set of human-snail population/infection data across all demographics could allow for detailed local calibration based on algebraic relations between state variables and model parameters (for example, see Gurarie et al., 2010, Gurarie et al., 2016). In the case of incomplete data sets, one has to fill in missing demographic/snail inputs by comparison with other, better sampled communities. In the present analysis, the CWRU model employed such methodology for Mozambique communities using some biological parameters previously calibrated from data from another *S. haematobium* control study in Kenya (the Msambweni study, Gurarie et al., 2015). This aspect of the model is described in full in Supplementary Information 2.

### 2.3 Key model differences

Model development was conducted separately and based on the details of the *S. haematobium* life cycle and known population processes (Anderson and May, 1991). A summary of differences between the models is outlined in Table 1. Some parameter values were derived from the published literature, such as the adult worm life expectancy and the density-dependent relationship between worm burden and egg output, whilst others were derived through parameter estimation based on observed epidemiological pattern as recorded in age stratified age-intensity and age prevalence curves. This was conducted separately by the two groups but based on a common source of data; namely, a cluster-randomized trial of MDA-based control of *S. haematobium* infection in Mozambican communities. The two models are similar in their account of human host demography and an age-dependent force of infection resulting in a characteristic age profile of infection. Both models can be traced back to an approach studied by Kostitzin (1934) in which the parasite burden is described in terms of the number of hosts with a given number of worms. Processes of parasite acquisition and death then determine the dynamics of the number of hosts with a given number of worms.

The two models can be broken down into two basic sub-models; a description of the parasite burden within the hosts and a model of the dynamics of infectious material outside the host in the environment and within the snail vector. Both models have significant differences in each of these areas (see relevant supplementary information files for model details). In the examples shown below, the parameter values match those used to fit to the baseline of the Copuito data, since both models achieve a reasonable fit for this data.

The two within-host models diverge in their treatment of the distribution of parasites across the population. The CWRU model has no explicitly prescribed worm distribution and instead makes the worm burden strata $\left\{h_{m}\left(t\right)\right\}$ dynamic variables (termed stratified worm burden model, SWB, for this reason). The model treats all hosts of a given age as having the same force of infection. Analysis of the set of equations governing the worm burden strata show an approximately Poisson distribution of worms over the host population, with additional heterogeneity arising from age dependent effects. The ICL model includes an additional source of heterogeneity, drawing contact rates from a gamma distribution and leading to a NB worm burden across the host population with fixed (estimated) aggregation parameter *k*. Such distributions of schistosomes have been observed in autopsy data (Cheever, 1968).

**Fig. 1:**
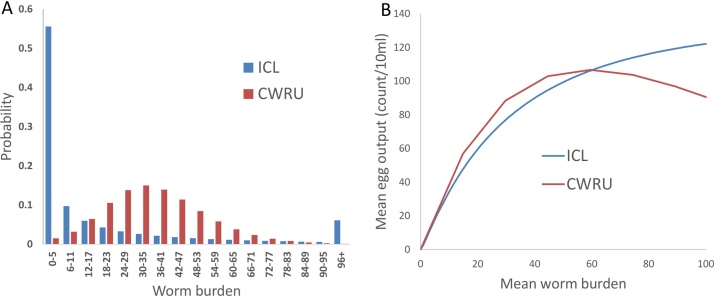
Details of the two within-host models. A) Distribution of worm burdens among hosts for the two models. Bin sizes match the stratification of worm burdens within the CWRU model. B) Mean measureable egg output as a function of mean worm burden. (parameter values based on Copuito fit).

A key difference between the two sub-models is in the degree of heterogeneity among hosts. The mean host burden in the two models are of the same order, with ICL having a mean of 22 worms per person and CWRU having approximately 36 worms (Fig. 1A). Fig. 1B shows that the relationship between mean worm burden and egg output is very similar in the two cases, implying that the CWRU model generates more egg output at equilibrium. The different approaches to host heterogeneity lead to quite different worm burden distributions. While the ICL model gives a highly over-dispersed distribution, the CWRU model gives a more Poisson-like distribution with greater aggregation. This difference has several potential consequences. Firstly, the two models are liable to give different assessments of infection prevalence for the same mean worm burden. For the ICL model, the prevalence is a function of the mean worm burden and aggregation parameter of the negative binomial distribution. The prevalence is known to be insensitive to changes in mean worm burden when prevalence is high (Anderson et al., 2014). For the CWRU model, the prevalence (fraction of persons shedding eggs) is a function of mean intensity and aggregation parameter of the negative binomial distribution assumed for the egg release process from mated worms. This function rather saturates at high intensity values. A second consequence, the assessment of morbidity, is discussed in the conclusion.

For the environmental part of the two models, there are also considerable differences. In the CWRU environmental sub-model, the dynamics of the snail population are described by a combined growth-SEI transmission model, using a 3-component system of ODEs. The ICL sub-model is based on a very simple description of infectious material in the environment. Material is expelled into the environment from the hosts and is subject to linear decay over time. This simplification is justified on the basis of timescale arguments; the timescale of infectious processes in snails is much shorter than that of the worms’ lifespans in the host and therefore merit a much less detailed description. However, it should also be noted that the CWRU approach potentially introduces new nonlinearities and types of behaviour not present in the ICL one. Additionally, any treatment interventions that include an element of molluscicide can be explicitly modelled within the CWRU framework, but only approximated in the ICL model.

**Fig. 2:**
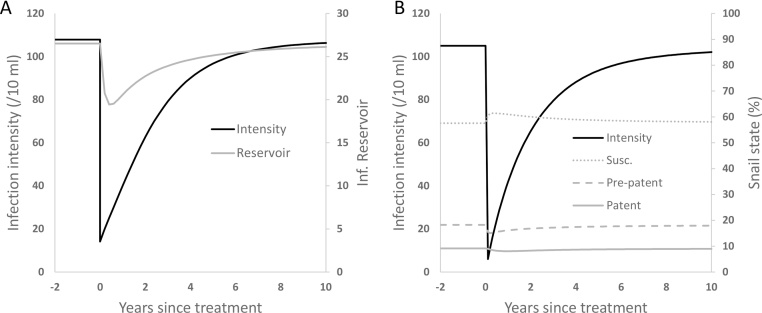
Recovery (bounce-back) from single round of treatment (parameter values based on Copuito fit with 86% coverage assumed among SAC for both models to facilitate the comparison). A) ICL model, showing egg intensity for school-age children and reservoir status against time. B) CWRU model, showing egg intensity for school-age children and the three variables of the snail infection sub-model as percentages of the total population.

Fig. 2 shows the bounce back from a single round of treatment in both models assuming 86% coverage of SAC. Fig. 2A shows the response of egg output in school-aged children and in the infectious reservoir state in the ICL model. Fig. 2B shows egg output and susceptible, pre-patent and patently infected snails in the CWRU model. The infection intensity profiles are broadly similar, quantitatively and qualitatively, with the exception that the initial rate of recovery is higher in the CWRU model. The response of the environmental sub-model is clearly different. For the ICL model, the reservoir responds within a few months with a 20% drop and then recovers over the next 5 years. The CWRU snail model is less sensitive to the drop in egg output showing 12% drop in patent snail prevalence over ten months. Since the reservoir state and the patent snail population govern the underlying force of infection experienced by hosts in the community, the CWRU model effectively has a constant force of infection, at least in response to a single round of treatment focusing on SAC.

### 2.4 Data

The data used for calibration and testing of the models were taken from a multi-centre randomized trial of *S. haematobium* control performed in Cabo Delgado Province from 2011 to 2015. This community-based trial, performed by the Schistosomiasis Control Initiative and the Catholic University of Mozambique with the support of the Schistosomiasis Consortium for Operational Research and Evaluation (SCORE), examined the relative impact of community- vs. school-based drug delivery and the effects of yearly vs. every other year treatment over a four year period (Ezeamama et al., 2016). Impact of treatment was assessed by testing a random sample of one hundred 9–12 year olds in each community at baseline and 12 months after each round of treatment. For the present model comparison exercise, individual-level data from two different villages were used to calibrate and test the models. These were selected from Arm 4 of the parent control study for which MDA was given as school-based therapy each year for four years. During each treatment cycle, children who were not enrolled in school were encouraged to participate and the total number of treatments delivered to SAC was recorded by the programme team.

The transmission models were fitted to data coming from individual villages, on the assumption that the village is taken to be the smallest independent disease transmission unit. A number of data quality issues arose during our processing of the data for this purpose. In particular, these involved uncertainty about exact ages in a region that does not have birth records and uncertainty about the size of the target population. In order to approximate programme treatment coverage (doses delivered to SAC/total SAC), population estimates for resident SAC were derived as a 30% fraction of the total population, developed by extrapolation of the 2007 Mozambique government population census figures to the programme’s starting year, 2011. School enrollment was determined to be low in these villages (30–40%). It was also apparent that in-school children had a consistently higher rate of MDA participation than out-of-school SAC.

In addition, several irregularities became evident during our analysis of the available data. For example, in some village data sets, there was an increase in the intensity and/or infection prevalence after an initial round of treatment, clearly indicating transient effects such as migration or immigration that are not accounted for by the two models due to lack of robust information on these effects. Such villages were excluded and from those remaining, the villages of Catambo and Copuito were chosen as they were exhibiting the typical epidemiological patterns of *S. haematobium* infection. Refer to Table 2 for a summary of the data used to fit the models.

### 2.5 Parameter fitting methodology

#### 2.5.1 ICL model

A parameter direct fit to the Mozambique data proved impossible due to the inconsistencies and missing data described above. Instead more reliable data from a prior study were used to provide an age profile and parameters through a Markov Chain Monte Carlo (MCMC) fit. The model was then successfully fitted to baseline Mozambique egg intensity data in the 9–12 year age group by varying R_0_.

However, the available treatment coverage data is more problematic. It was immediately clear from the data in Table 2 that given the high efficacy of praziquantel against *S. haematobium*, the quoted coverage in year 1 is insufficient to account for the drop in mean intensity between year 1 and 2. The data shows that coverages are calculated using the total number of SAC as the denominator, as estimated from a census, not those who attended school. Since it is unclear what the correct denominator should be, its value was estimated from the data from year 1 and 2 with the value carried forward to calculate the coverage for treatment in year 3. This value was then used to split the SAC model population into two sub-populations within the model; one representing children within the programme, receiving treatment and the other outside of the school system, not being treated.

The fitted model was simulated through the second round of treatment to give a prediction for the intensity of infection among 9–12 year olds within the treatment programme at the year 3 time point, which could then be compared to the observed value in each village.

#### 2.5.2 CWRU model

The human part of the coupled human-snail system consists of 4 demographic groups; Pre-SAC (children), SAC (school age attending school), SACN (school age not attending school), and O (old/adult). Groups SAC, SACN are known fractions of total school-age pool S, and it was assumed they are identical in terms of risk/exposure and their in-host biology. Hence, SAC and SACN contribute equally to transmission. The only difference between the two was that SAC were subject to regular MDA/monitoring, while SACN remained out of reach.

The general strategy of the calibration scheme was that unknown model parameters were estimated using a Bayesian statistical approach (see Supplementary Information 2 for details). Specifically, school-based egg test data from two consecutive years (baseline year and the first follow up year) were used to specify four unknown model parameters relating to snail prevalence and reproduction, relative contact rates, and within host relative worm fecundity of different age groups with respect to the SAC group. Furthermore, baseline intensity and infection prevalence data of the SAC group were used in intermediate dynamic steps to specify within-host worm fecundity and rate of worm accumulation for this age group and consequently for other age groups using relative values of fecundity and contact rates.

The CWRU model then used a Markov Chain Monte Carlo (MCMC) approach to draw samples from the posterior distributions of unknown parameters. To validate the model and fitting procedures, posterior distributions of unknown parameters were used to generate an ensemble of SAC prevalence time series by running dynamic MDA simulations through year 3 and incorporating the influence of treatment coverage in year 1 and 2. Model infection prevalence estimates and their credible intervals in year 3 were then compared with the observed SAC prevalence. The credible intervals reflect the confidence level for prevalence estimates given the data and the model. A summary of parameters and values for both models can be found in Table 3.

## 3 Results and discussion

For both groups, the models were used to attempt to predict the infection prevalence and intensity of the host population in year 3, for two villages, Catambo and Copuito. Data from the first two years were used for parameterization of the models.

**Fig. 3:**
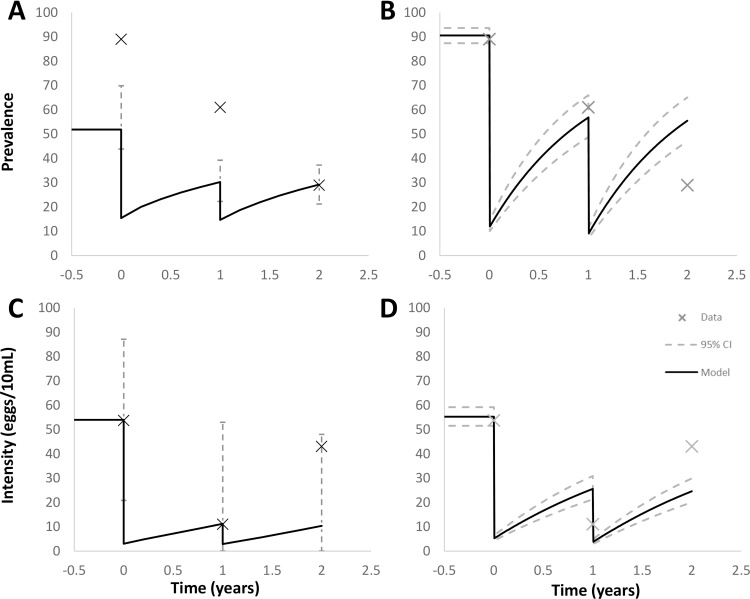
Catambo model fits and predictions. A) ICL model fit and predictions for year 3 egg prevalence including 95% predictive interval for the measured prevalence across the sample of approximately 100 individuals. Model fitted to corresponding egg intensity data. B) CWRU model fit and predictions for year 3 prevalence. Broken lines represent 95% credible intervals due to parameter uncertainty. C) ICL model fit and predictions for year 3 egg intensity including 95% predictive interval for the mean measured egg counts across the sample of approximately 100 individuals. D) CWRU model fit and predictions for year 3 intensity. Broken lines represent 95% credible intervals due to parameter uncertainty.

**Fig. 4:**
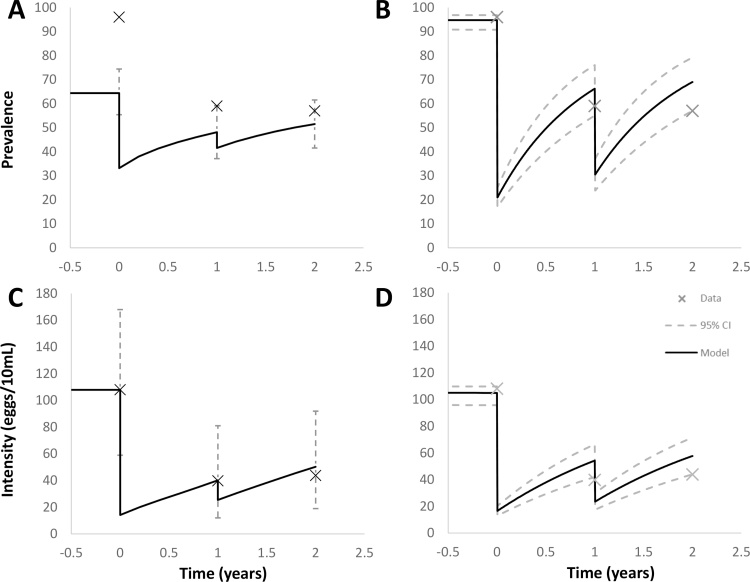
Copuito model fits and predictions. **A**) ICL model fit and predictions for year 3 egg prevalence including 95% predictive interval for the measured prevalence across the sample of approximately 100 individuals. Model fitted to corresponding egg intensity data. B) CWRU model fit and predictions for year 3 prevalence. Broken lines represent 95% credible intervals due to parameter uncertainty. C) ICL model fit and predictions for year 3 egg intensity including 95% predictive interval for the mean measured egg counts across the sample of approximately 100 individuals. D) CWRU model fit and predictions for year 3 intensity. Broken lines represent 95% credible intervals due to parameter uncertainty.

The ability of the ICL model to match the observed data differs markedly between the two villages studied (see Figs. 3 C and 4 C ). In the case of Copuito village, the projection for year 3 mean egg/10 mL urine matches the observed data very well. The fitted value for coverage of SAC captured within the treatment programme is approximately 86%. A value this high is well within the expectation of a well-organised school-based control programme.

The error bars in Figs. 3 C and 4 C reflect the 95% predictive interval for the model’s prediction of the mean egg count, given a sampled population matching that which underlies the data. The high degree of uncertainty arises from two sources; uncertainty as to how many worms individuals harbour for a given mean female worm burden and the variability across measurements in egg count per 10 ml, given a particular number of worms. Within the ICL model formulation, both of these uncertainties are described by negative binomial distributions and hence compound to give a negative binomial with a very high variance to mean ratio and low *k* value. This suggests that predictions in terms of mean measured egg outputs will generally not be very precise (although they may be accurate within a given sample) unless very large samples are involved including multiple sampling of the same urine collection from an individual. Figs. 3 A and 4 A shows the predictions of the ICL model for the evolution of prevalence, given a model fitted to egg intensity data. The fit to the data is clearly quite poor in general. For Copuito, prevalence at the end of years 1 and 2 are just within the predictive interval of the model, but the baseline is far from the model estimate. The model behaviour is also characterised by much smaller changes in prevalence in response to treatment than the CWRU model (see below). This relative insensitivity to changes in underlying worm burden is a feature of the negative binomial distribution, as already discussed.

The CWRU model is calibrated using school-based SAC prevalence data from the two villages, which assumes that these reported data are unbiased estimates and that the reported coverage data estimate coverage among the school-attending SAC group. By neglecting small coverage levels in other age groups and among non-school attenders, the CWRU model used the first two rounds to calibrate unknown model parameters and predict prevalence among school-attending SAC in year 3.

The CWRU approach gives a reasonable prevalence estimate among school-attending SAC in year 3 for Copuito at 0.69 (CI: 0.57-0.79) compared to 0.57, although it largely overestimates the corresponding prevalence for Catambo at 0.55 (CI: 0.47-0.65) compared to 0.29 (see Figs. 3 B and 4 B). The CWRU fitting procedure takes the complete egg-test distribution data, rather than specific test statistics (mean intensity, prevalence et al). While it assumed no coverage outside SAC school-attenders, the results show a similar pattern to the ICL model results, namely, good predictions for Copuito but underestimating intensity level in year 3 for Catambo (see Figs. 3 D and 4 D).

In the case of Catambo village, the model predictions for the year 3 mean egg intensity are very different from the observed values. The fitted coverage value in this case is approximately 95% and that for the second round approximately 80%, leading to a predicted intensity of around 10 eggs/10 mL, in contrast to an observed value of 43 eggs/10 mL. However, the increase in intensity in this village between year 2 and 3, by a factor of 4, is difficult to understand. For a parasite with a relatively long lifespan, rates of change of parasite burden would be far too slow to allow a bounce back close to the equilibrium value within a single year

A possible explanation is that the make-up of the sampled population has changed to include a previously untreated, heavily infected group in year 3. As is apparent from data quality issues discussed in the Methods section, the data available was frequently insufficient to clearly assess what proportion the treated SAC represented within the community as a whole (uncertain denominators) and whether those aspects of the treatment programme may have changed as the programme proceeded year by year. Were the same individuals treated each year, or were they different? Sampling from the untreated, non-school attending school-aged population would dramatically increase the measured mean infection intensity among 9–12 year olds. Given the structure of the school-based treatment programme, this may well have occurred. This highlights the importance of collecting precise data on who is treated and who is sampled post-treatment.

The ICL group used the first two rounds of egg intensity data in part to estimate what coverage level was most likely each year to give the observed pattern. While this approach leads to a reasonable fit for predictions for Copuito in year 3, more reliable data on coverage should be informing the estimation of the biological parameters of the model, rather than the other way around.

**Fig. 5:**
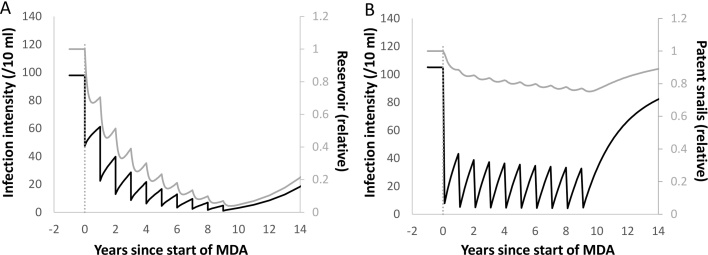
Impact of 10 years of MDA, focused on SAC with 75% coverage for A) the ICL model and B) the CWRU model. Back lines show the egg intensity in the SAC age group and grey lines indicate relative mean force of infection experienced by hosts, being A) the relative status of the infectious reservoir and B) the relative abundance of patent snails.

Fig. 5 compares the response of the two models to long term targeted MDA. A program of 10 years of MDA targeted at SAC is simulated and the figure shows the impact on intensity (eggs/10 ml) and on the mean force of infection experienced by hosts as indicated by the infectious reservoir (ICL) and the patent snail population (CWRU), respectively. The decline in egg output is markedly greater for the ICL model than for the CWRU model and is primarily a result of the small change in force of infection in the latter model. In the ICL model, infectious reservoir status and hence FOI drops by more than 90% over the 10 year intervention and elimination appears likely within the next 10 years. The response in the CWRU model is consolidated within a couple of years and remains constant throughout the remainder of the intervention. Bounce back post-treatment is also much faster for the CWRU model for the same reason. Initial response to treatment is also informative of model dynamics. Given a linear relationship between worms and egg output, a drop of around 70% in egg output would be expected. The response in egg output of the ICL model to the first round of treatment is closer to 50%. The drop in worm burden increases the net egg output of the remaining worms, a result of the density dependent fecundity which is responsible for the model’s stable endemic equilibrium. In the case of the CWRU model, the drop in egg output exceeds the 70%. This indicates that the endemic equilibrium in this model is not a product of density dependent fecundity of parasites in the host. It almost certainly reflects the non-linear impact of the lack of mating pairs of worms in hosts after treatment leading to lower egg production than would be expected from the loss of worms alone.

The ICL group feel that, particularly when dealing with data of poor quality, egg intensity data has certain advantages over prevalence. Although more parameters are required to link to the basic worm-centred dynamic model (the egg output per female worm, or ideally a full model of the egg count diagnostic protocol), it reduces the reliance on prevalence data that is very dependent on the source and quality of population size information and the magnitude of the negative binomial aggregation parameter, *k*. In the current dataset, population data often came from entirely different sources to infection data, such as censuses rather than the detailed demography of the study villages, and the process of calculating prevalence compounds the biases in the numerator and denominator rather than reducing them.

Different egg-test outputs can be employed in model calibration, depending on its structure, including mean intensity, prevalence and/or other statistics. For the ICL model mean test intensity provides the natural input. The CWRU model employs complete egg-test data via simulated “random egg release” by SWB groups/communities. A combined “mean and prevalence” data may seem advantageous compared to any one choice. However, it’s not clear (it would require additional study to determine) how much independent information and uncertainty is contained in a typical test dataset.

## 4 Conclusion

The key model differences identified in the methods section account for some of the differences observed between the two models. The CWRU model has a noticeably faster bounce back from infection than the ICL model and this is almost certainly a consequence of the nonlinear force of snail infection. The ICL environmental model has a larger and relatively long-lasting response to the drop in worm burden. The fits to the Copuito data suggest that a more responsive environmental sub-model gives a more accurate disease bounce back. These differences also suggest that the models would not come to the same conclusions about the possibility of elimination, with the ICL model allowing easier elimination. This conclusion is demonstrated by the impact of MDA shown in Fig. 5. The differences in the responses of the force of infection to long-term treatment lead to qualitatively different results, in terms of the impact on egg output during treatment, the probability of elimination and the speed of bounce back after the intervention is interrupted.

The negative binomial distribution with fixed aggregation of the ICL model causes estimates of prevalence based on fitting to intensity data to generally underestimate at the baseline and also to underestimate the change generated by treatment (See Fig. 3, Fig. 4A). However, as already discussed, apparent problems with the accuracy of the prevalence data may be partly responsible for this effect. A further important aspect of the large difference in variance between the two models is predictions of morbidity. There is a very large discrepancy in the prevalence of high burden individuals between the two models, with the CWRU having a great deal less. Given that morbidity is primarily a consequence of very high worm burdens and the associated high production of eggs, the two models will give highly divergent pictures of the amount of morbidity predicted and likely lead to different conclusions as to the cost effectiveness of any regime of treatment. It is a feature of these models, then, that the description of prevalence is closely linked to the ability of the model to account for morbidity in a community. Such differences between models clearly need to be analysed and resolved if models are to be used to inform policy. Direct observations of worm burden is not possible; what data there is (from animal models and post-mortem in humans) suggest aggregated distributions (Cheever, 1968, Crombie and Anderson, 1985). The different relationships between prevalence and intensity in the two models and their different responses to treatment should be resolvable given high quality matching prevalence/intensity data across a treated age group. Differences in force of infection across a number of years of MDA are potentially detectable by long-term monitoring of the abundance of infectious snails.

The gathering of reliable monitoring and evaluation (M&E) data from national treatment programmes is clearly an expensive and time-consuming undertaking. Any attempt to create a definitive record of patterns of treatment and monitoring will always be undermined by issues of migration, seasonal movements of labour and issues of compliance. However, the authors suggest that a few key pieces of data would improve the capacity of models to offer accurate descriptions of disease progress and help in the design of drug coverage programmes to eliminate parasite transmission or morbidity in children and adults.

A key requirement for data that is to be used with models forecasting outcome scenarios, after a defined treatment programmes is accurate denominator information on the population sizes in various groupings such as school-aged children, those attending school, and village population size. This impacts assessment of coverage rates directly, but is also essential for any attempt to model the dynamics of prevalence over time and under treatment. Often, national treatment programmes do not routinely collect egg intensity data across different age groups. As such, prevalence is the only epidemiological data collected longitudinally. Model predictions can only be as reliable as the data that informs them, therefore, detailed denominator data is key. This is one of the limitations of present-day MDA programmes (see review by Shuford et al., 2016). The collection of egg intensity data, while more labour intensive, helps to reduce the need for accurate prevalence data. Access to programme protocols and reports on implementation, if available, would be extremely useful in interpreting these often large and complex datasets on the impact of MDA on schistosome prevalence and intensity across different age classes.

Effective treatment coverage levels will be key in achieving the goals of WHO-recommended preventive chemotherapy (PCT) programmes (World Health Organization, 2006, World Health Organization, 2012). The WHO’s NTD Strategic and Technical Advisory Group recommends a systematic programme of M&E surveys of both population size and treatment uptake, in order to provide a clear distinction between flaws in implementation (low coverage) vs. the greater biological risk of persistent infection and reinfection. Currently, in many regions of endemic infection, these guidelines are not well adhered to. We hope the work presented here will help to highlight the fundamental importance of careful and accurate data collection to inform modelling studies as part of ongoing efforts to improve the design, monitoring and implementation of NTD control programmes.

## Competing interests

Roy Anderson is a Non-Executive Director of GlaxoSmithKline (GSK). GlaxoSmithKline played no role in study design, data collection and analysis, decision to publish, or preparation of the manuscript.

## Author contributions

All authors contributed equally.

## Figures and Tables

**Table 1:** Model comparison.

Model structure	ICL model	CWRU model
Simulation framework	Deterministic-probabilistic	Deterministic-probabilistic
Implementation language	R	Mathematica/Python
Age-related exposure to infection	Relative exposure estimated from age-stratified infection intensity data	Baseline test data provides relevant SAC parameters, the other (untested) younger/older age groups are estimated via community dynamic simulations (Y1-Y3)
Distribution of individual predisposition to heavy or light infection	No individual-level propensity	Age-specific biological (in-host) parameters: worm fecundity, egg-release aggregation
Age-related contribution to the reservoir of infection in the environment	Assumed to be identical to exposure to infection	Age-specific exposure/contamination, and the resulting human-snail transmission coefficients
Distribution of contributions to reservoir of infection among individuals	No individual-level contributions	No individual contribution, but age-specific contributions
Acquired immunity to infection	Assume no acquired immunity	Assume no acquired immunity
Coverage and compliance	Structured by age group	Structured by age group
Demographic data source	Data collected in SCORE project	Data collected in SCORE project
Reproductive model	Mating function for dioecious monogamous parasite	Mating count for dioecious monogamous parasite, with age-specific fecundity and crowding. Low cut-off worm burden for mating success

**Table 2:** Mean egg intensity, prevalence and coverage data and population sizes used to parameterise the models.

Mean intensity (eggs/10 mL) ages 9–12 years	Catambo	Copuito
Year 1 (eggs/10 mL)	53.8	108
Year 2 (eggs/10 mL)	11	40
Year 3 (eggs/10 mL)	43	43.8

Prevalence (%)		
Year 1	89	96
Year 2	61	59
Year 3	29	57

School-based treatment coverage (%)		
Year 1	57	50
Year 2	42	22

Sample sizes within school-aged children		
Year 1	85	73
Year 2	82	51

**Table 3:** Parameter values for *Schistosoma haematobium* used in making the model predictions.

Parameter	ICL model	CWRU model
Transmission intensity	R_0_ = 1.6*	No BRN is employed in model formulation, and infection intensity is formulated via estimated (human-snail) transmission coefficients
Level of aggregation of parasites in host population	Negative binomial, k = 0.24	No worm distribution pattern is prescribed, but arises naturally through dynamic worm-strata variables
Aggregation parameter for the distribution of repeated eggs counts	Not used in the current analysis	Egg-release is Negative binomial with prescribed (estimated) aggregation k=.01-.05
Density-dependent fecundity	Exponential density dependence (γ=0.005)	Exponential E^ (-w/100), for worm burden w
Adult worm life expectancy, L	4 years	4-5 years
Praziquantel drug efficacy	94%	80%
Relative contribution/exposure to environmental reservoir of infection	0.3* (ages 0-5), 1* (ages 5-10), 0.05* (ages 10 + )	Estimated values Ages 0-5: .5-.6 Ages 15 +: .45-.5
Average survival of infectious agents in environmental stage	4 months	Determine by snail population/infection dynamics with prepatent period = 2 month
Prepatent period	None	2-months for snails, none for humans
Female worm fecundity	5.2 egg/worm/10 mL specimen	30 egg/worm/10 mL specimen

*Indicates derived from parameter fitting.
