# Supplementary material for: A comparison of two mathematical models of the impact of mass drug administration on the transmission and control of schistosomiasis
Source: Epidemics. 2017 Mar;18:29–37. doi: 10.1016/j.epidem.2017.02.003 (PMC5340850; doi:10.1016/j.epidem.2017.02.003)
Supplement: Supplementary file 1 [file mmc1.docx]

**A comparison of two mathematical models of the impact of mass drug administration on the transmission and control of schistosomiasis**

Truscott JE, Gurarie D, Alsallaq R, Yoon N, Farrell SH, Turner HC, Phillips AE, Aurelio HO, Ferro J, Toor J, King CH & Anderson RM

**Supplementary information 2: The CWRU Model**

**A: Structured SWB system**

In a single (homogeneous) SWB system host population is divided into worm burden strata $\left\{h_{m}\right\}$ $\sum_{m} h_{m}=1$, partitioned with worm-step $\Delta w\geq1$: $m \Delta w\leq w<\left(m+1\right)\Delta w$ for $h_{m}$. The worm-step serves as hypothetical *mating threshold*, so $h_{0}$ are infection-free (no mated couples), while $\left\{h_{m}:m\geq1\right\}$ carry mated couples, whose number is estimated from binomial sex-ratio (Anderson-May 91)

The transitions among strata are determined by human force of infection (FOI) $\lambda$ (= rate of worm accumulation/$\Delta w$), resolution rates $\gamma_{k}=k \gamma$($\gamma$ - mean worm mortality), and population turnover rate $\mu$ (mortality, maturation, migration, etc.), see Figure 1

Figure 1: SWB strata and transitions

Source terms $\vec{S}=\left(S_{k}\right)$ in Figure 1 represent demographic inputs from younger to older age-group. For child group its source $\vec{S}=\left(\mu,0,0,...\right)$, comes into zero stratum only, as all newborns are infection-free.

Structured SWB consists of multiple population groups each one represented by its own SWB. In our case, **C, S, O** –groups have relative population sizes $H_{C}+H_{S}+H_{O}=1$, and variables $\vec{h}_{i}\left(t\right)=\left\{h_{0}^{i},h_{1}^{i},...\right\}$, $i=\left(C,S,O\right)$ obey coupled differential system with tridiagonal transition matrix $M\left(\lambda,\mu,\gamma\right)$

$$\begin{matrix} \frac{d \vec{h}^{C}}{dt}=M\left(\lambda_{C},\mu_{C},\gamma_{C}\right)\cdot\vec{h}^{C}+\vec{S}_{C}=\mu_{C}\delta_{0} \\ \frac{d \vec{h}^{S}}{dt}=M\left(\lambda_{S},\mu_{S},\gamma_{S}\right)\cdot\vec{h}^{S}+\mu_{S}\vec{h}^{C} \\ \frac{d \vec{h}^{O}}{dt}=M\left(\lambda_{A},\mu_{A},\gamma_{A}\right)\cdot\vec{h}^{O}+\mu_{A}\vec{h}^{S} \end{matrix}$$

**B. Random egg-release** and **human infectivity**

Egg release by mated females and individual hosts depends on worm fecundity $\rho_{m}$, and mated-couple count (for $h_{m}$ -stratum). The former is given by crowding function $\rho_{m}=\rho_{0} \zeta^{m}$, with maximal value $\rho_{0}$and loss factor $0<\zeta<1$. The predicted egg-release by $h_{m}$-hosts, , gives its mean (expected) value used as measure of host infectivity. The actual release should be random (NB) with mean $E_{m}$ and aggregation .


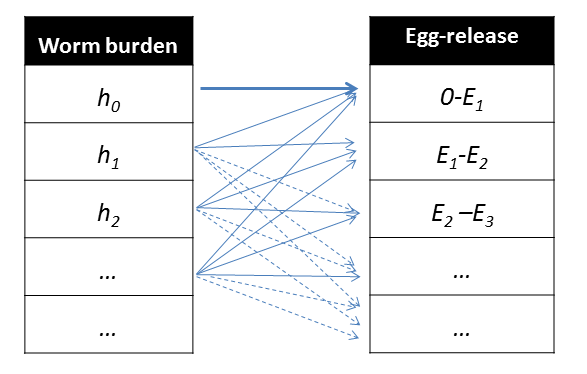


Figure 2: Distributed egg release by SWB strata

Individual egg-counts-counts by all SWB hosts (strata $\left\{h_{m}\right\}$) generate a mixed NB-distribution, illustrated in the schematic plot above,

Each simulated egg-test of SWB community is then a random sample of size *H* (sampled pool) drawn from the mixture distribution $P$. Host infectivity for each SWB age-group is determined by its mean egg release (= mean $P$ )

; $i=\left(C,S,O\right)$

Egg-test prevalence for $P$ is computed via

**C. Snail population and transmission dynamics**

*Dynamic variables* for the snail model are population densities (per unit habitat) *x*- susceptible; *y*- prepatent; *z*-patent; $N=x+y+z$ - total (Figure 3)

$$\to^{\beta\left(1-N/K\right)} \mathop{x}\limits_{\begin{matrix} \downarrow\\ \nu\end{matrix}} \rightleftarrows_{\left(1-c\right)r}^{\Lambda} \mathop{y}\limits_{\begin{matrix} \downarrow\\ \nu\end{matrix}} \to^{c r} \mathop{z}\limits_{\begin{matrix} \downarrow\\ \nu\end{matrix}}$$

Figure 3: Schematic snail model

Combined growth-SEI transmission is described by 3 differential equations

$$\begin{matrix} \frac{dx}{dt}=\beta\left(1-\frac{N}{K}\right)\left(x+y\right)-\left(\Lambda+\nu\right)x +r\left(1-c\right)y \\ \frac{dy}{dt}=\Lambda x-\left(r+\nu\right) y \\ \frac{dz}{dt}=c r y-\nu z \end{matrix}$$

Basic processes and inputs of snail model include i) snail reproduction (logistic growth) $\beta\left(x+y\right)\left(1-N/K\right)$, with maximal reproduction rate $\beta$ and carrying capacity *K*; ii) snail mortality $\nu$; iii) snail FOI $\Lambda$(determined by human egg release) ; iv) recovery rate *r* (prepatency period = 1/*r*); iv) patency conversion fraction $0<c<1$ (Figure 3). Only susceptible and prepatent snails ($x+y$) can reproduce.

Parameter values and ranges for the snail system are given in Table 2. For stationary environment (*K* – constant), variables$x,y,z,N$ can be rescaled relative to *K,* so that total density $N=1-\nu/\beta$, in the absence of transmission, $\Lambda=0$ .

Snail FOI $\Lambda$ is determined by relative human infectivities $E_{i}$ , population fractions $H_{i}$ and transmission coefficients $B_{i}$. They produce combined miracidium release by all host groups

The resulting snail FOI depends on mean “miracidia per snail” number $M/N$ , as

$$\Lambda=\Lambda_{0}\left(1-e^{-M/N}\right)$$

i.e. the product of “miracidium invasion rate$\Lambda_{0}$” times “probability of invasion” (assuming Poisson distribution of miracidia).

Human FOI depend on patent snail prevalence *z*, and transmission coefficients $A_{i}$

$$\lambda_{C}=A_{C} z; \lambda_{S}=A_{S} z; \lambda_{O}=A_{O} z$$

Two sets of equations - coupled by FOI $\lambda_{i}\left(z\right)$ , and $\Lambda\left(\vec{h}_{C},\vec{h}_{S},\vec{h}_{O}\right)$ , make up a coupled human-snail system.

**D. Endemic equilibria of coupled human-snail systems**

Endemic equilibria of SWB system can be computed numerically by inverting transition matrices

$M\left(\lambda,\mu,\gamma\right)$ . The resulting distributions depend on human FOI, demographic turnover, worm mortality inputs $\lambda_{i},\mu_{i},\gamma_{i}$ ,

$$\vec{h}_{C}\left(\lambda_{C}|\mu_{C},\gamma_{C}\right); \vec{h}^{S}\left(\lambda_{S},\lambda_{C}|..\right); \vec{h}_{O}\left(\lambda_{O};\lambda_{S};\lambda_{C}|..\right)$$

Equilibrium solution of snail equations gives patent snail density$z^{*}$ as function of FOI $\Lambda$ and biological parameters $\beta,\nu,r,c$ , namely,

$z^{*}=\frac{\left\{\left(1-\frac{\nu+c r}{\beta}\right)\Lambda-\frac{\nu\left(r+\nu\right)}{\beta}\right\}\Lambda}{\left(\Lambda+r+\nu\right)\left\{\left(1+\frac{\nu}{cr}\right)\Lambda+\frac{\nu}{cr}\left(r+\nu\right)\right\}}$ ;

The range of variable $z^{*}$ (used in calibration) is determined by snail biological parameters

$0<z^{*}<\frac{1-\left(\nu+c r\right)/\beta}{1+\nu/(c r)}$;

Equilibrium population density $N^{*}$ of system is reduced due to $\Lambda$

$$N^{*}\left(\Lambda\right)=1-\frac{\nu}{\beta}-\frac{r c \Lambda}{\beta\left(\Lambda+r+\nu\right)}>0$$

Condition $N^{*}\left(\Lambda\right)>0$ imposes a constraint on parameter values $\frac{\nu+c r}{\beta}<1$ .

Typical equilibrium patterns for the coupled human-snail system for n=20 strata are shown in Figure 4


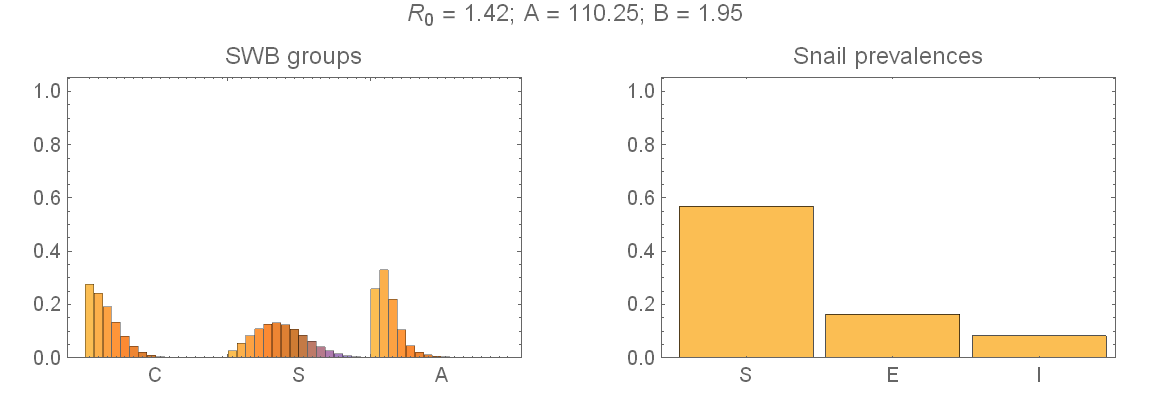


Figure 4:Equilibrium solutions of (C-S-A) SWB system coupled (0.1) to snail coupled to snail system (0.3) via transmission coefficients - (human-to-snail, and - snail-to human.

Table 1: Summary of model parameters

|  | Parameters (known, fixed) | | Calibrated | |
| --- | --- | --- | --- | --- |
| Human | Age-specific fecundity  Crowding ^(1)^  Egg- NB aggregation ^(1)^ | $\rho_{0}^{C}=\rho_{0}^{S}=\rho; \rho_{0}^{O}=\tilde{\rho} \rho$ $\begin{matrix} \zeta=.91 \\ k=.05 \end{matrix}$ | SAC fecundity:  Relative “adult/SAC” fecundity | $<\rho<$ $0<\tilde{\rho}<1$ |
| Snail^(1)^ | $\begin{matrix} \nu=2/\text{year} \\ r=10/\text{year} \\ c=.05-.2 \\ \Lambda_{0}=12-15/\text{year} \end{matrix}$ | | Proliferation | $\beta=12-16/\text{year}$ |
| Transmission | Contacts and transmission $\left(A_{i},B_{i}\right)$ for $i=\left(C,S,O\right)$ Behavioral age pattern/contacts | | SAC  Relative contact | $A=A_{S};B=B_{S}$ $\omega_{C}; \omega_{O}$ |

^(1)^ Based on Kenyan calibration (Gurarie et al 2016)

**E. Baseline (endemic) calibration of transmission coefficients**

The coupled human-snail system is made of 4 demographic groups **C** (child), **SA** (school age attending school), **SN** (school age not attending school), **O** (old/adult). School-age groups **SA, SN** are known fractions of the total pool **S.** The basic parameters are transmission coefficients: $A_{i}$ (snail-to-human) and $B_{i}$ (human-to-snail) $i=\mathbf{C},\mathbf{S},\mathbf{O}$, relating human/snail infectivity to their respective FOI (**SA,SN** have identical $A_{S},B_{S}$) . Transmission coefficients are proportional to relative exposure (contact rates): “child /adult over SAC”, $\omega_{C};\omega_{O}<1$, and relative worm fecundity “adult/SAC”, $\tilde{\rho}<1$. Specifically,

The goal of baseline calibration is to estimate $A_{i},B_{i}$ using SAC egg-test data over two years of study. The estimates involve 5 undetermined parameters: human triplet $\theta_{H}=\left(\omega_{C},\omega_{O},\tilde{\rho}\right)$, and 2 snail inputs $\theta_{S}=\left(z^{*},\beta\right)$- equilibrium patent snail density, and its maximal replication rate. The relations between transmission coefficients $\left(A_{i},B_{i}\right)$ and 5 calibrated parameters

$$\theta=\left(\omega_{C},\omega_{O},\tilde{\rho}, z^{*},\beta\right)$$

is explained below. Calibration proceeds in 2 steps:

1. *endemic step* employs egg-count data on Y1 (mean intensity $E$ and prevalence $P$) along with a particular of $\theta$ to estimate $A_{i},B_{i}$ as functions of $\left(P,E,\theta\right)$
2. *dynamic step* takes estimated $A_{i},B_{i}$ of step 1 and simulates a 3-year MDA control history of the host community. The SAC prevalence outputs for Y1-Y3 along with test data, are then used to assign likelihood of $\theta$

Specifically, transmission coefficients of 3 age groups are expressed through a single pair $A,B$ of SAC, and the latter are given by the above functions of calibrated parameters $\theta$ and data $D=\left(E_{D},P_{D}\right)$

$$\begin{matrix} A\left(\theta|D\right)=\frac{\lambda^{*}\left(D\right)}{z^{*}} \\ B\left(\theta|D\right)=\frac{-\ln\left(1-\Lambda^{*}/\Lambda_{0}\right) N^{*}\left(\Lambda^{*}\right)}{E\left(\theta|D\right)} \end{matrix}$$

Auxiliary functions used in this step include i) SAC FOI $\lambda^{*}\left(D\right)$, ii) snail FOI $\Lambda^{*}\left(\beta,z\right)$, iii) relative host infectivity $E\left(\theta|D\right)$ . Having estimated SAC transmission rates $A,B$ we get other groups $A_{C},B_{C};A_{O},B_{O}$ via equations . We shall derive them below

1. Human FOI $\lambda^{*}$ and maximal fecundity $\rho^{*}$ are computed from an algebraic system of equations for prevalence-intensity derived from hypothesized NB egg release

For a given test data (prevalence intensity) $P_{D}$ , $E_{D}$, and fixed “crowding, aggregation” parameters $\zeta,k$ (adopted from [Gurarie et al 2016]) we solve numerically equations

$$\begin{matrix} P\left(\lambda,\rho|\zeta,k\right)=P_{D} \\ E\left(\lambda,\rho|\zeta\right)=E_{D} \end{matrix}$$

to get equilibrium estimates of$\lambda^{*};\rho^{*}$ in terms of data values

$$\lambda^{*}\left(D\right)=\lambda^{*}\left(E_{D},P_{D}\right)$$

1. Snail FOI $\Lambda^{*}\left(\beta,z\right)$ is derived by solving equilibrium equation for $\Lambda$ with prescribed $z=z^{*}$, followed by equilibrium snail density $N^{*}\left(\Lambda^{*}\right)$ estimated via .
2. Relative human infectivity function ( = “mean egg release/fecundity”)

$E\left(\lambda|\zeta\right)=\sum_{m} h_{m}\left(\lambda\right)\zeta^{m}$ ,

is applied to 3 age groups to get age-specific infectivities expressed through a single parameter - SAC $\lambda^{*}=\lambda_{S}^{*}$ and relative contacts rates $\omega_{i}$

$$E_{C}=E\left(\omega_{C}\lambda^{*}\right); E_{S}=E\left(\lambda^{*}\right); E_{O}=E\left(\omega_{O}\lambda^{*}\right)$$

Functions produce a combined (community mean) host infectivity

$$E\left(\theta|D\right)=\omega_{C}H_{C}E_{C}+H_{S}E_{S}+\omega_{O}\rho_{O}H_{O}E_{O}$$

Functions $\lambda^{*}\left(D\right)$ , $\Lambda^{*}\left(\beta,z\right)$, $N^{*}\left(\Lambda^{*}\right)$ ,$E\left(\theta|D\right)$ make up transmission coefficients $A\left(\theta|D\right)$ , and $B\left(\theta|D\right)$ of the calibration step 1.

**F. Dynamic Bayesian calibration**

For dynamic calibration we use the following data:

1. Infectious prevalence among school-attending children (SA) in years: 1 (baseline), and 2.
2. Endemic intensity among school-attending children (in year 1).
3. PZQ coverage among school-attending children in years: 1, and 2.
4. Sample sizes of school-attending children in years 1 and 2.

The general framework of Bayesian analysis to estimate probability distributions for the uncertain parameters given the model and available data, $\mathbf{D}$. If $\mathbf{\theta}$is the set of parameters to be estimated and$M(\mathbf{\theta})$is the mathematical model (coupled H-S system), and $\mathbf{\psi}=M(\mathbf{\theta})$ - the projected model outputs (school-attending SAC prevalence values on Y1-Y2). If we are able to specify a likelihood function $p(\mathbf{D}|\mathbf{\psi})$ for the probability of data given the model parameter values (or the corresponding prevalence values), then the posterior distribution of $\mathbf{\theta}$given the model and data is given by Bayes theorem:

$$p(\mathbf{\theta}|\mathbf{D},\mathbf{\psi})\propto p(\mathbf{\theta})p(\mathbf{D}|\mathbf{\psi})$$

For the likelihood function we used the combined likelihood of prevalence data values $\mathrm{D}_{1},D_{2}$ in years 1 and 2. We used binomial distribution that naturally incorporates the number of surveyed school-aged persons ($N_{s,t}$ ) in each year:

Where $N_{s,t}$, $\mathrm{D}_{t}$ and $\psi_{t}$ are respectively the sample size and the data and model prevalence in year $t$.

Using the equations -- we can evaluate the posterior density function $p(\mathbf{\theta}|\mathbf{D},\mathbf{\psi})$. We use the Markov Chain Monte Carlo (MCMC) approach to sample from the posterior distribution.

In our setup vector ($\mathbf{\theta}$) of five model parameters is drawn from uniform distributions within the following ranges

Table : Priors on uncertain parameters

| Parameter | Uniform range |
| --- | --- |
| $z$ | 0.01 to 0.17 |
| $\beta$ | 3 to 25 |
| $\omega_{C}$ | 0.1 to 0.99 |
| $\omega_{O}$ | 0.1 to 0.99 |
| $\tilde{\rho}$ | Abs(0.5-$\omega_{O}$) |
